# Crystal structure of a rare trigonal bipyramidal titanium(IV) coordination complex: tri­chlorido­(3,3′-di-*tert*-butyl-2′-hy­droxy-5,5′,6,6′-tetra­methyl-1,1′-biphenyl-2-olato-κ*O* ^2^)(tetra­hydro­furan-κ*O*)­titanium(IV)

**DOI:** 10.1107/S2056989016020156

**Published:** 2017-01-01

**Authors:** Yun Young Kim, Joseph M. Tanski

**Affiliations:** a Department of Chemistry, Vassar College, Poughkeepsie, NY 12604, USA

**Keywords:** crystal structure, coordination complex, titanium in trigonal–bipyramidal coordination

## Abstract

The title compound, [Ti(C_24_H_33_O_2_)Cl_3_(C_4_H_8_O)], is a rare example of a trigonal–bipyramidal titanium coordination complex with three chloride and two oxygen donor ligands. The asymmetric unit contains two independent mol­ecules having essentially the same conformation. The mol­ecules feature the titanium(IV) metal cation complexed with three chloride ligands, a tetra­hydro­furan mol­ecule, and one oxygen atom from the resolved ligand precursor (*R*)-(+)-5,5′,6,6′-tetra­methyl-3,3′-di-*t*-butyl-1,1′-biphenyl-2,2′-diol, where the remaining phenolic hydrogen atom engages in inter­molecular O—H⋯Cl hydrogen bonding. In one mol­ecule, the THF ligand is disordered over two orientations with refined site occupancies of 0.50 (3).

## Abstract 2

The synthesis and structure of a rare example of a trigonal–bipyramidal titanium coordination complex with three chlorides and two oxygen donor ligands is reported.

## Chemical context   

Asymmetric Lewis acid catalysis with titanium coordination compounds featuring chiral ligands for the selective synthesis of resolved small mol­ecule organic compounds is a well established field of chemistry (Ramón & Yus, 2006 ▸). Chiral diol ligands such as 1,1′-bi-2-naphthol (BINOL) and 2,2-dimethyl-α,α,α′,α′-tetra­phenyl-1,3-dioxolane-4,5-di­methanol (TADDOL) are two ligand types that have seen frequent use (Baker-Salisbury *et al.*, 2014 ▸). In work aimed at preparing new titanium asymmetric Lewis acid catalysts, the title compound was obtained as a crystalline solid from tetra­chlorido­bis(tetra­hydro­furan)­titanium(IV) and the BINOL ligand (*R*)-(+)-5,5′,6,6′-tetra­methyl-3,3′-di-*t*-butyl-1,1′-biphenyl-2,2′-diol (BIPHEN). The complex, [BIPHEN-*κ* ^1^ *O*]TiCl_3_(THF), is a rare example of a trigonal–bipyramidal coordination geometry for titanium(IV), with a Chemical Bonding Classification (CBC) designation of Ti*LX*_4_ (Green, 1995 ▸).
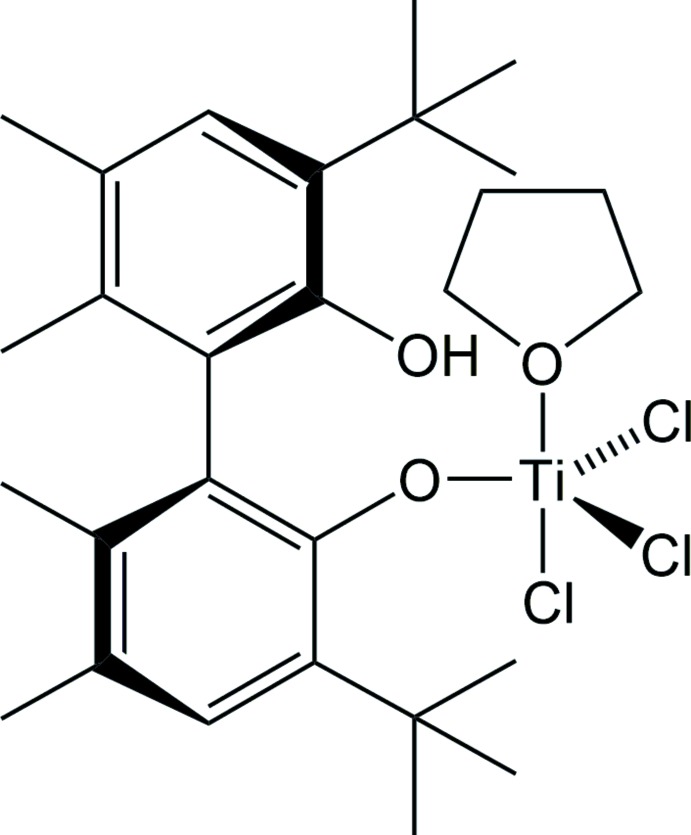


## Structural commentary   

**Figure 1:**
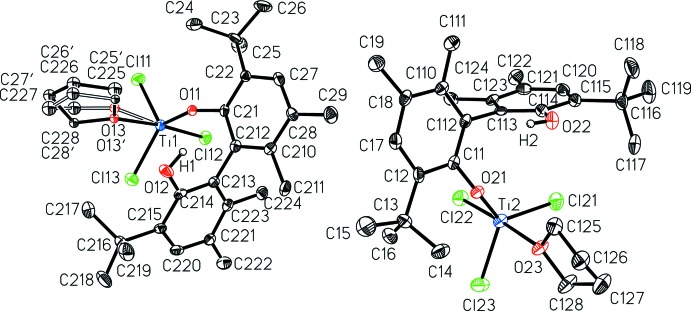
A view of the two independent mol­ecules of [BIPHEN-*κ* ^1^ *O*]TiCl_3_(THF) with the atom-numbering scheme. Displacement ellipsoids are shown at the 50% probability level. Hydrogen atoms on carbon have been removed for clarity.

The asymmetric unit of the title compound, [BIPHEN-*κ* ^1^ *O*]TiCl_3_(THF), contains two independent mol­ecules (Fig. 1 ▸), the only notable difference being the twofold disorder of the tetra­hydro­furan ligand on Ti2. The trigonal–bipyramidal mol­ecules have very similar metrical parameters. The BIPHEN phenoxide distances, Ti1—O11 of 1.767 (4) Å and Ti2—O21 of 1.756 (4) Å are similar, and shorter than the Ti—O bonds to the neutral coordinating tetra­hydro­furan (THF), with Ti1—O13 2.157 (9), Ti1—O13′ 2.112 (9), and Ti2—O23 2.125 (4) Å. The THF occupies an axial position in the trigonal–bipyramidal complex, while the BIPHEN phenoxide is equatorial. The other axial position contains chloride with distances of Ti1—Cl12 2.2728 (17) Å and Ti2—Cl22 2.2685 (18) Å. The remaining two equatorial sites are occupied by chlorides with similar Ti—Cl bond lengths (see Supporting Information). The complex is very nearly trigonal–bipyramidal, with linear axial O—Ti—Cl angles O13—Ti1—Cl12 174.5 (4)°, O13′—Ti1—Cl12 173.9 (4)° and O23—Ti2—Cl22 176.50 (13)°. The angles in the trigonal plane are further away from the ideal 120°, for example O11—Ti1—Cl13 131.13 (13)°, O11—Ti1—Cl11 113.94 (13)°, Cl13—Ti1—Cl11 113.72 (7)°, while the axial-equatorial angles are all quite near 90°. The absolute structure parameters confirm the *R* axial chirality of the BIPHEN ligand, with Flack *x* = 0.03 (2) and Hooft *y* = 0.03 (2) (Dolomanov *et al.*, 2009 ▸).

## Supra­molecular features   

**Figure 2:**
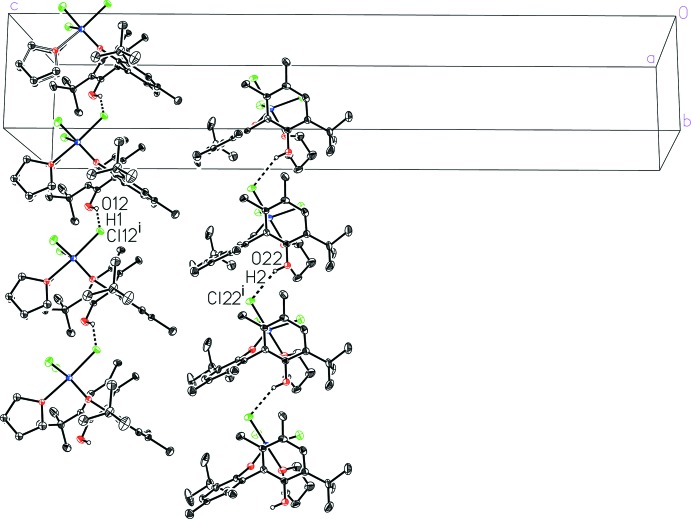
A view of the inter­molecular hydrogen bonding in [BIPHEN-*κ* ^1^ *O*]TiCl_3_(THF) (dashed lines). Displacement ellipsoids are shown at the 50% probability level. Hydrogen atoms on carbon have been removed for clarity.

The mol­ecules pack together in the solid state *via* van der Waals forces and hydrogen bonding between the phenolic OH groups and chloride ligands on neighboring mol­ecules, O12—H1⋯Cl12^i^ and O22—H2⋯Cl22^i^ [symmetry code: (i) *x*, *y* + 1, *z*] with H⋯Cl distances of 2.62 (4) and 2.59 (4) Å, respectively (Table 1 ▸). These inter­actions create zigzag chains linking equivalent mol­ecules extending parallel to the *b* axis (Fig. 2 ▸).

## Database survey   

The Cambridge Structural Database (Groom *et al.*, 2016 ▸) contains one related titanium BIPHEN structure and a few five-coordinate titanium complexes with three chloride and two oxygen donor ligands. The structure of BIPHEN(TiCl_3_)_2_ comprises TiCl_3_ moieties additionally coordinated by each phenoxide O atom of the ligand (Chisholm *et al.*, 2003 ▸). A very similar structure to the title compound, [(EMind)O]TiCl_3_(THF), also has a bulky phenoxide ligand in an equatorial position and THF in an axial position on the trigonal–bipyramid (Kanazawa *et al.*, 2016 ▸). Also similar, the same trigonal–bipyramidal arrangement is seen in a complex with two TiCl_3_(ethyl acetate) units coordinated by phenoxides derived from the diol 2,2′-(1,3-butadiyne-1,4-di­yl)bis­[phenol] (Saied *et al.*, 1998*a* ▸). The structure of 4,4′-di­methyl­benzo­phenone coordinated to TiCl_3_ with the bis­(phenoxide) derived from a fluorenediol also contains titanium in a trigonal–bipyramidal coordination environment; however, all three chlorides are in the equatorial plane and the ketone and phenoxide are axial (Saied *et al.*, 1998*b* ▸). A dinuclear disilane-1,2-diolateoxo-bridged titanium complex (Krempner *et al.*, 2007 ▸) exhibits two unique distorted trigonal–bipyramidal coordination environments, while a trinuclear mandelic acid methyl ­ester moiety exhibits two distorted trigonal–bipyramidal coordination environments and a penta­gonal–bipyramidal seven-coordinate titanium (Ziemer *et al.*, 2008 ▸).

## Synthesis and crystallization   

Under a nitro­gen atmosphere, tetra­chlorido­bis­(tetra­hydro­furan)­titanium(IV) (23.4 mg, 0.07 mmol) was added to (*R*)-(+)-5,5′,6,6′-tetra­methyl-3,3′-di-*t*-butyl-1,1′-biphenyl-2,2′-diol (50 mg, 0.14 mmol) in C_6_H_6_ (2.5 ml) and the benzene was allowed to slowly evaporate yielding red plate crystals within seven days. The synthesis could be scaled up and the material collected by filtration, yielding a dark-red crystalline powder; however, the material quickly powders into a pink amorphous solid upon loss of coordinating THF, as observed by ^1^H NMR of the decomposition product.

## Refinement   

Crystal data, data collection and structure refinement details are summarized in Table 2 ▸. H atoms on carbon were included in calculated positions and refined using a riding model with C—H = 0.95, 0.98 and 0.99 Å and *U*_iso_(H) = 1.2, 1.5 and 1.2*U*_eq_(C) of the aryl, methyl and methyl­ene C atoms, respectively. The position of the phenolic hydrogen atoms were found in the difference map and the atom refined semi-freely using a distance restraint *d*(O—H) = 0.84 Å, and with *U*_iso_(H) = 1.2*U*_eq_(O).

## Supplementary Material

Crystal structure: contains datablock(s) global, I. DOI: 10.1107/S2056989016020156/rz5202sup1.cif

Structure factors: contains datablock(s) I. DOI: 10.1107/S2056989016020156/rz5202Isup2.hkl

CCDC reference: 1523643

Additional supporting information: crystallographic information; 3D view; checkCIF report

## Figures and Tables

**Table 1:** Hydrogen-bond geometry (Å, °)

*D*—H⋯*A*	*D*—H	H⋯*A*	*D*⋯*A*	*D*—H⋯*A*
O12—H1⋯Cl12^i^	0.85 (3)	2.62 (4)	3.333 (4)	143 (5)
O22—H2⋯Cl22^i^	0.84 (3)	2.59 (4)	3.350 (4)	153 (5)

Symmetry code: (i) 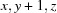.

**Table 2:** Experimental details

Crystal data	
Chemical formula	[Ti(C_24_H_33_O_2_)Cl_3_(C_4_H_8_O)]
*M*_r_	579.86
Crystal system, space group	Monoclinic, *P*2_1_
Temperature (K)	125
*a*, *b*, *c* (Å)	10.289 (4), 7.141 (3), 40.330 (16)
β (°)	95.164 (7)
*V* (Å^3^)	2951 (2)
*Z*	4
Radiation type	Mo *K*α
μ (mm^−1^)	0.59
Crystal size (mm)	0.31 × 0.11 × 0.01

Data collection	
Diffractometer	Bruker APEXII CCD
Absorption correction	Multi-scan (*SADABS*; Bruker, 2013 ▸)
*T*_min_, *T*_max_	0.81, 0.99
No. of measured, independent and observed [*I* > 2σ(*I*)] reflections	76791, 17956, 10069
*R*_int_	0.156
(sin θ/λ)_max_ (Å^−1^)	0.716

Refinement	
*R*[*F* ^2^ > 2σ(*F* ^2^)], *wR*(*F* ^2^), *S*	0.064, 0.124, 1.00
No. of reflections	17956
No. of parameters	653
No. of restraints	3
H-atom treatment	H atoms treated by a mixture of independent and constrained refinement
Δρ_max_, Δρ_min_ (e Å^−3^)	0.56, −0.55
Absolute structure	Flack *x* determined using 3136 quotients [(*I* ^+^)−(*I* ^−^)]/[(*I* ^+^)+(*I* ^−^)] (Parsons *et al.*, 2013 ▸)
Absolute structure parameter	0.03 (2)

Computer programs: *APEX2* and *SAINT* (Bruker, 2013 ▸), *SHELXT2014* (Sheldrick, 2015*a* ▸), *SHELXL2014* (Sheldrick, 2015*b* ▸), *SHELXTL* (Sheldrick, 2008 ▸), *OLEX2* (Dolomanov *et al.*, 2009 ▸) and *Mercury* (Macrae *et al.*, 2008 ▸).

## supplementary crystallographic information

### Crystal data

**Table:**

[TiCl_3_(C_24_H_33_O_2_)(C_4_H_8_O)]	*F*(000) = 1224
*M**_r_* = 579.86	*D*_x_ = 1.305 Mg m^−^^3^
Monoclinic, *P*2_1_	Mo *K*α radiation, λ = 0.71073 Å
*a* = 10.289 (4) Å	Cell parameters from 9897 reflections
*b* = 7.141 (3) Å	θ = 2.3–28.9°
*c* = 40.330 (16) Å	µ = 0.59 mm^−^^1^
β = 95.164 (7)°	*T* = 125 K
*V* = 2951 (2) Å^3^	Plate, red
*Z* = 4	0.31 × 0.11 × 0.01 mm

### Data collection

**Table:**

Bruker APEXII CCD diffractometer	17956 independent reflections
Radiation source: fine-focus sealed tube	10069 reflections with *I* > 2σ(*I*)
Graphite monochromator	*R*_int_ = 0.156
Detector resolution: 8.3333 pixels mm^-1^	θ_max_ = 30.6°, θ_min_ = 1.5°
φ and ω scans	*h* = −14→14
Absorption correction: multi-scan (SADABS; Bruker, 2013)	*k* = −10→10
*T*_min_ = 0.81, *T*_max_ = 0.99	*l* = −57→57
76791 measured reflections	

### Refinement

**Table:**

Refinement on *F*^2^	Hydrogen site location: mixed
Least-squares matrix: full	H atoms treated by a mixture of independent and constrained refinement
*R*[*F*^2^ > 2σ(*F*^2^)] = 0.064	*w* = 1/[σ^2^(*F*_o_^2^) + (0.037*P*)^2^] where *P* = (*F*_o_^2^ + 2*F*_c_^2^)/3
*wR*(*F*^2^) = 0.124	(Δ/σ)_max_ = 0.001
*S* = 1.00	Δρ_max_ = 0.56 e Å^−^^3^
17956 reflections	Δρ_min_ = −0.55 e Å^−^^3^
653 parameters	Absolute structure: Flack *x* determined using 3136 quotients [(*I*^+^)-(*I*^-^)]/[(*I*^+^)+(*I*^-^)] (Parsons *et al.*, 2013)
3 restraints	Absolute structure parameter: 0.03 (2)

### Special details

**Table:**

Geometry. All esds (except the esd in the dihedral angle between two l.s. planes) are estimated using the full covariance matrix. The cell esds are taken into account individually in the estimation of esds in distances, angles and torsion angles; correlations between esds in cell parameters are only used when they are defined by crystal symmetry. An approximate (isotropic) treatment of cell esds is used for estimating esds involving l.s. planes.

### Fractional atomic coordinates and isotropic or equivalent isotropic displacement parameters (Å^2^)

**Table:**

	*x*	*y*	*z*	*U*_iso_*/*U*_eq_	Occ. (<1)
Ti1	0.15076 (9)	0.06244 (13)	0.90044 (2)	0.0147 (2)	
Ti2	0.60744 (10)	0.57807 (14)	0.62786 (3)	0.0200 (2)	
Cl11	−0.04160 (13)	−0.0389 (2)	0.91666 (3)	0.0259 (3)	
Cl12	0.16052 (14)	−0.17155 (19)	0.86255 (4)	0.0226 (3)	
Cl13	0.32527 (13)	−0.0434 (2)	0.93240 (3)	0.0239 (3)	
Cl21	0.53928 (16)	0.4972 (2)	0.57555 (4)	0.0317 (4)	
Cl22	0.49421 (15)	0.3477 (2)	0.65118 (4)	0.0270 (4)	
Cl23	0.80166 (14)	0.4650 (2)	0.65011 (4)	0.0320 (4)	
O11	0.1354 (3)	0.2425 (5)	0.87027 (9)	0.0136 (8)	
O12	0.3958 (4)	0.5492 (6)	0.89644 (9)	0.0224 (9)	
H1	0.339 (4)	0.579 (9)	0.8809 (10)	0.027*	
O21	0.5361 (4)	0.7615 (5)	0.64884 (9)	0.0185 (9)	
O22	0.3647 (4)	1.0640 (6)	0.59083 (9)	0.0219 (9)	
H2	0.381 (5)	1.110 (8)	0.6098 (8)	0.026*	
O23	0.7241 (4)	0.7845 (5)	0.60685 (10)	0.0247 (10)	
C11	0.4538 (5)	0.8656 (7)	0.66688 (13)	0.0173 (12)	
C12	0.5005 (5)	0.9402 (8)	0.69815 (13)	0.0204 (13)	
C13	0.6393 (6)	0.9084 (9)	0.71423 (15)	0.0294 (15)	
C14	0.7450 (5)	0.9710 (10)	0.69140 (15)	0.0313 (15)	
H14A	0.8313	0.9642	0.7038	0.047*	
H14B	0.7277	1.1001	0.684	0.047*	
H14C	0.7428	0.8883	0.672	0.047*	
C15	0.6658 (7)	1.0211 (13)	0.74656 (16)	0.061 (3)	
H15A	0.7582	1.0101	0.7546	0.091*	
H15B	0.6119	0.972	0.7634	0.091*	
H15C	0.6442	1.153	0.7423	0.091*	
C16	0.6578 (6)	0.6995 (11)	0.72326 (17)	0.0438 (19)	
H16A	0.7472	0.6787	0.7331	0.066*	
H16B	0.642	0.6233	0.7031	0.066*	
H16C	0.5961	0.6638	0.7393	0.066*	
C17	0.4077 (5)	1.0378 (8)	0.71449 (13)	0.0213 (13)	
H17A	0.435	1.0898	0.7357	0.026*	
C18	0.2776 (5)	1.0652 (8)	0.70211 (13)	0.0197 (12)	
C19	0.1860 (6)	1.1667 (8)	0.72264 (15)	0.0300 (15)	
H19A	0.1504	1.2764	0.7104	0.045*	
H19B	0.2332	1.2072	0.7436	0.045*	
H19C	0.1146	1.083	0.7274	0.045*	
C21	0.1589 (5)	0.3449 (7)	0.84236 (12)	0.0132 (11)	
C22	0.0537 (5)	0.4116 (7)	0.82072 (13)	0.0143 (12)	
C23	−0.0905 (5)	0.3701 (8)	0.82497 (14)	0.0239 (14)	
C24	−0.1298 (6)	0.4392 (9)	0.85904 (14)	0.0298 (15)	
H24A	−0.0841	0.3647	0.8769	0.045*	
H24B	−0.2242	0.4252	0.8598	0.045*	
H24C	−0.1058	0.5713	0.862	0.045*	
C25	−0.1147 (6)	0.1584 (8)	0.82152 (16)	0.0319 (16)	
H25A	−0.0637	0.0925	0.8396	0.048*	
H25B	−0.0883	0.1154	0.8001	0.048*	
H25C	−0.2077	0.1324	0.8227	0.048*	
C26	−0.1797 (5)	0.4689 (11)	0.79820 (15)	0.0386 (17)	
H26A	−0.1676	0.6047	0.8003	0.058*	
H26B	−0.2708	0.4374	0.801	0.058*	
H26C	−0.158	0.4282	0.7762	0.058*	
C27	0.0896 (6)	0.5126 (7)	0.79334 (13)	0.0194 (13)	
H27A	0.0218	0.5622	0.7783	0.023*	
C28	0.2184 (5)	0.5457 (8)	0.78653 (12)	0.0173 (12)	
C29	0.2458 (6)	0.6567 (8)	0.75601 (14)	0.0279 (15)	
H29A	0.303	0.584	0.7427	0.042*	
H29B	0.2887	0.7748	0.7629	0.042*	
H29C	0.1635	0.6831	0.7427	0.042*	
C110	0.2357 (5)	0.9910 (7)	0.67039 (13)	0.0174 (12)	
C111	0.0941 (5)	1.0074 (8)	0.65711 (14)	0.0248 (14)	
H11A	0.0606	1.1304	0.6629	0.037*	
H11B	0.0433	0.9091	0.6669	0.037*	
H11C	0.0866	0.9934	0.6328	0.037*	
C112	0.3253 (5)	0.8912 (7)	0.65281 (13)	0.0164 (12)	
C113	0.2798 (5)	0.8024 (7)	0.61991 (13)	0.0146 (12)	
C114	0.3005 (5)	0.8944 (7)	0.59019 (13)	0.0170 (12)	
C115	0.2562 (5)	0.8200 (8)	0.55867 (13)	0.0192 (12)	
C116	0.2827 (6)	0.9166 (8)	0.52556 (14)	0.0265 (14)	
C117	0.4299 (6)	0.9452 (10)	0.52335 (14)	0.0340 (16)	
H11D	0.4616	1.0448	0.5387	0.051*	
H11E	0.4454	0.9805	0.5006	0.051*	
H11F	0.4763	0.8285	0.5294	0.051*	
C118	0.2144 (7)	1.1076 (9)	0.52238 (16)	0.0396 (18)	
H11G	0.2497	1.1896	0.5405	0.059*	
H11H	0.1205	1.0908	0.5237	0.059*	
H11I	0.2295	1.1643	0.5009	0.059*	
C119	0.2323 (7)	0.7983 (10)	0.49540 (15)	0.0417 (19)	
H11J	0.274	0.6748	0.4969	0.063*	
H11K	0.2532	0.8609	0.4749	0.063*	
H11L	0.1376	0.7835	0.4951	0.063*	
C120	0.1870 (5)	0.6522 (8)	0.55951 (14)	0.0219 (13)	
H12B	0.1554	0.598	0.5388	0.026*	
C121	0.1610 (5)	0.5587 (8)	0.58838 (14)	0.0210 (12)	
C122	0.0827 (6)	0.3803 (8)	0.58648 (15)	0.0275 (15)	
H12C	0.0106	0.3911	0.6007	0.041*	
H12D	0.139	0.2753	0.5941	0.041*	
H12E	0.0476	0.3583	0.5634	0.041*	
C123	0.2093 (5)	0.6331 (7)	0.61928 (14)	0.0161 (12)	
C124	0.1825 (5)	0.5346 (8)	0.65082 (13)	0.0234 (13)	
H12F	0.2362	0.5899	0.6696	0.035*	
H12G	0.2038	0.4015	0.649	0.035*	
H12H	0.0901	0.5482	0.6544	0.035*	
C125	0.6836 (6)	0.9795 (8)	0.60020 (15)	0.0264 (14)	
H12I	0.6573	1.04	0.6206	0.032*	
H12J	0.6096	0.9851	0.5828	0.032*	
C126	0.8018 (6)	1.0726 (9)	0.58865 (15)	0.0305 (14)	
H12K	0.8628	1.1126	0.6077	0.037*	
H12L	0.7776	1.1825	0.5745	0.037*	
C127	0.8605 (7)	0.9180 (9)	0.56885 (17)	0.0390 (18)	
H12M	0.8155	0.9086	0.5462	0.047*	
H12N	0.9547	0.9398	0.5672	0.047*	
C128	0.8390 (6)	0.7464 (9)	0.58863 (17)	0.0360 (17)	
H12O	0.8229	0.637	0.5738	0.043*	
H12P	0.9164	0.7201	0.6044	0.043*	
C210	0.3203 (5)	0.4727 (7)	0.80815 (13)	0.0154 (11)	
C211	0.4600 (5)	0.4962 (8)	0.80030 (13)	0.0225 (13)	
H21A	0.5187	0.4596	0.8197	0.034*	
H21B	0.4757	0.6275	0.7948	0.034*	
H21C	0.4763	0.4169	0.7813	0.034*	
C212	0.2898 (5)	0.3722 (7)	0.83678 (13)	0.0150 (12)	
C213	0.3973 (5)	0.2860 (7)	0.85896 (13)	0.0152 (12)	
C214	0.4484 (5)	0.3793 (7)	0.88810 (13)	0.0154 (12)	
C215	0.5524 (5)	0.3068 (7)	0.90883 (13)	0.0159 (12)	
C216	0.6054 (5)	0.4046 (8)	0.94181 (13)	0.0194 (13)	
C217	0.4962 (6)	0.4228 (9)	0.96536 (14)	0.0300 (15)	
H21D	0.4268	0.5034	0.9551	0.045*	
H21E	0.5319	0.478	0.9865	0.045*	
H21F	0.4605	0.2986	0.9695	0.045*	
C218	0.7130 (6)	0.2885 (9)	0.96042 (15)	0.0350 (17)	
H21G	0.679	0.1641	0.9652	0.052*	
H21H	0.7427	0.351	0.9814	0.052*	
H21I	0.7863	0.2759	0.9467	0.052*	
C219	0.6617 (6)	0.5977 (8)	0.93538 (15)	0.0297 (15)	
H21J	0.5917	0.6809	0.9262	0.044*	
H21K	0.7278	0.5864	0.9194	0.044*	
H21L	0.7019	0.6498	0.9563	0.044*	
C220	0.6074 (5)	0.1392 (7)	0.89813 (14)	0.0190 (13)	
H22B	0.6794	0.0877	0.9115	0.023*	
C221	0.5627 (5)	0.0441 (8)	0.86906 (13)	0.0172 (12)	
C222	0.6268 (6)	−0.1334 (7)	0.85938 (14)	0.0227 (13)	
H22C	0.6464	−0.1253	0.8361	0.034*	
H22D	0.568	−0.2392	0.8621	0.034*	
H22E	0.708	−0.1519	0.8737	0.034*	
C223	0.4542 (5)	0.1158 (7)	0.84961 (13)	0.0169 (12)	
C224	0.4013 (5)	0.0151 (7)	0.81800 (13)	0.0218 (13)	
H22F	0.3165	0.0685	0.81	0.033*	
H22G	0.3909	−0.1184	0.8227	0.033*	
H22H	0.4624	0.0303	0.8009	0.033*	
O13	0.1609 (13)	0.2791 (12)	0.9378 (2)	0.011 (2)*	0.50 (3)
C225	0.0961 (19)	0.463 (2)	0.9323 (4)	0.023 (4)*	0.50 (3)
H22A	0.147	0.5434	0.9182	0.028*	0.50 (3)
H22I	0.0074	0.4464	0.921	0.028*	0.50 (3)
C226	0.0890 (18)	0.549 (2)	0.9649 (4)	0.020 (4)*	0.50 (3)
H22J	0.1623	0.6374	0.9698	0.024*	0.50 (3)
H22K	0.006	0.6183	0.9656	0.024*	0.50 (3)
C227	0.0964 (18)	0.3992 (17)	0.9888 (3)	0.017 (3)*	0.50 (3)
H22L	0.0077	0.3643	0.9943	0.02*	0.50 (3)
H22M	0.1476	0.4394	1.0095	0.02*	0.50 (3)
C228	0.1629 (17)	0.2306 (15)	0.9735 (3)	0.011 (3)*	0.50 (3)
H22N	0.2535	0.2149	0.9837	0.013*	0.50 (3)
H22O	0.1137	0.1137	0.9766	0.013*	0.50 (3)
O13'	0.1223 (14)	0.2678 (12)	0.9368 (2)	0.012 (2)*	0.50 (3)
C25'	0.1329 (16)	0.4767 (18)	0.9327 (3)	0.009 (3)*	0.50 (3)
H25D	0.2169	0.5098	0.924	0.011*	0.50 (3)
H25E	0.0608	0.5239	0.9171	0.011*	0.50 (3)
C26'	0.1253 (17)	0.559 (2)	0.9660 (4)	0.017 (3)*	0.50 (3)
H26D	0.1945	0.6542	0.9706	0.021*	0.50 (3)
H26E	0.0395	0.6196	0.9674	0.021*	0.50 (3)
C27'	0.1427 (19)	0.4070 (18)	0.9899 (3)	0.024 (4)*	0.50 (3)
H27B	0.0847	0.4251	1.0079	0.029*	0.50 (3)
H27C	0.2341	0.404	0.9999	0.029*	0.50 (3)
C28'	0.110 (2)	0.2295 (16)	0.9720 (3)	0.021 (3)*	0.50 (3)
H28A	0.1714	0.1291	0.9802	0.025*	0.50 (3)
H28B	0.0203	0.1897	0.9755	0.025*	0.50 (3)

### Atomic displacement parameters (Å^2^)

**Table:**

	*U*^11^	*U*^22^	*U*^33^	*U*^12^	*U*^13^	*U*^23^
Ti1	0.0174 (5)	0.0126 (4)	0.0144 (5)	0.0020 (4)	0.0022 (4)	0.0006 (4)
Ti2	0.0205 (6)	0.0159 (5)	0.0242 (6)	−0.0012 (5)	0.0056 (4)	−0.0011 (5)
Cl11	0.0196 (7)	0.0328 (8)	0.0262 (8)	0.0006 (7)	0.0063 (6)	0.0057 (7)
Cl12	0.0287 (8)	0.0172 (7)	0.0221 (8)	0.0018 (6)	0.0026 (6)	−0.0055 (6)
Cl13	0.0232 (8)	0.0240 (7)	0.0238 (8)	0.0021 (6)	−0.0016 (6)	0.0012 (7)
Cl21	0.0380 (9)	0.0324 (9)	0.0250 (8)	0.0008 (7)	0.0044 (7)	−0.0047 (7)
Cl22	0.0267 (9)	0.0213 (7)	0.0335 (9)	−0.0064 (7)	0.0054 (7)	0.0046 (7)
Cl23	0.0224 (8)	0.0300 (8)	0.0437 (9)	0.0024 (7)	0.0041 (7)	0.0068 (8)
O11	0.014 (2)	0.0145 (19)	0.012 (2)	0.0001 (15)	0.0020 (16)	0.0013 (15)
O12	0.024 (2)	0.019 (2)	0.022 (2)	0.0096 (19)	−0.0064 (17)	−0.0041 (19)
O21	0.017 (2)	0.018 (2)	0.021 (2)	0.0008 (16)	0.0048 (17)	0.0007 (17)
O22	0.030 (2)	0.0184 (19)	0.018 (2)	−0.007 (2)	0.0026 (18)	−0.001 (2)
O23	0.024 (2)	0.018 (2)	0.034 (3)	0.0026 (17)	0.015 (2)	0.0032 (18)
C11	0.021 (3)	0.017 (3)	0.015 (3)	−0.001 (2)	0.006 (2)	0.000 (2)
C12	0.024 (3)	0.025 (3)	0.012 (3)	−0.005 (3)	0.002 (2)	0.003 (3)
C13	0.020 (3)	0.047 (4)	0.021 (3)	0.003 (3)	−0.002 (3)	−0.003 (3)
C14	0.019 (3)	0.040 (4)	0.036 (4)	−0.003 (3)	0.008 (3)	0.000 (3)
C15	0.034 (4)	0.116 (8)	0.031 (4)	−0.009 (5)	−0.005 (3)	−0.037 (5)
C16	0.029 (4)	0.075 (5)	0.027 (4)	0.009 (4)	0.001 (3)	0.022 (4)
C17	0.025 (3)	0.024 (3)	0.016 (3)	−0.006 (3)	0.002 (2)	−0.001 (3)
C18	0.028 (3)	0.013 (2)	0.019 (3)	0.002 (3)	0.010 (2)	0.003 (3)
C19	0.032 (4)	0.031 (4)	0.027 (4)	0.009 (3)	0.004 (3)	−0.002 (3)
C21	0.022 (3)	0.007 (2)	0.011 (3)	0.005 (2)	0.004 (2)	0.000 (2)
C22	0.014 (3)	0.015 (3)	0.013 (3)	−0.001 (2)	0.001 (2)	−0.001 (2)
C23	0.014 (3)	0.034 (3)	0.022 (3)	0.001 (3)	−0.007 (3)	0.009 (3)
C24	0.022 (3)	0.035 (4)	0.032 (4)	0.009 (3)	0.004 (3)	0.004 (3)
C25	0.026 (4)	0.037 (4)	0.033 (4)	−0.015 (3)	0.002 (3)	−0.002 (3)
C26	0.014 (3)	0.060 (5)	0.041 (4)	0.004 (3)	0.000 (3)	0.017 (4)
C27	0.025 (3)	0.018 (3)	0.014 (3)	0.003 (2)	−0.002 (2)	0.001 (2)
C28	0.021 (3)	0.015 (3)	0.015 (3)	0.000 (2)	−0.001 (2)	0.003 (2)
C29	0.031 (4)	0.029 (3)	0.023 (3)	−0.006 (3)	0.003 (3)	0.005 (3)
C110	0.019 (3)	0.013 (3)	0.021 (3)	−0.001 (2)	0.007 (2)	0.003 (2)
C111	0.024 (3)	0.024 (3)	0.027 (3)	0.000 (3)	0.005 (3)	−0.002 (3)
C112	0.019 (3)	0.014 (3)	0.017 (3)	−0.004 (2)	0.006 (2)	0.001 (2)
C113	0.009 (3)	0.018 (3)	0.017 (3)	0.003 (2)	0.000 (2)	−0.001 (2)
C114	0.017 (3)	0.016 (3)	0.018 (3)	−0.002 (2)	0.000 (2)	−0.002 (2)
C115	0.020 (3)	0.020 (3)	0.018 (3)	0.001 (3)	0.002 (2)	0.000 (2)
C116	0.036 (4)	0.031 (3)	0.013 (3)	0.003 (3)	0.002 (3)	−0.004 (3)
C117	0.042 (4)	0.044 (4)	0.017 (3)	−0.001 (3)	0.009 (3)	0.005 (3)
C118	0.053 (5)	0.039 (4)	0.027 (4)	0.010 (3)	0.002 (3)	0.014 (3)
C119	0.057 (5)	0.049 (5)	0.019 (4)	−0.010 (4)	0.001 (3)	−0.001 (3)
C120	0.020 (3)	0.026 (3)	0.019 (3)	0.004 (3)	−0.002 (3)	−0.005 (3)
C121	0.017 (3)	0.017 (3)	0.029 (3)	0.001 (3)	0.001 (2)	−0.002 (3)
C122	0.028 (4)	0.028 (3)	0.026 (4)	−0.004 (3)	0.006 (3)	−0.009 (3)
C123	0.015 (3)	0.014 (3)	0.021 (3)	0.002 (2)	0.006 (2)	−0.002 (2)
C124	0.020 (3)	0.024 (3)	0.027 (3)	−0.003 (2)	0.000 (3)	0.006 (3)
C125	0.034 (4)	0.017 (3)	0.029 (3)	0.005 (3)	0.007 (3)	0.002 (3)
C126	0.035 (4)	0.021 (3)	0.037 (4)	−0.004 (3)	0.009 (3)	0.006 (3)
C127	0.041 (4)	0.035 (4)	0.044 (4)	0.001 (3)	0.023 (4)	0.006 (3)
C128	0.034 (4)	0.032 (4)	0.045 (4)	0.001 (3)	0.024 (3)	0.001 (3)
C210	0.016 (3)	0.012 (2)	0.019 (3)	−0.004 (2)	0.004 (2)	−0.002 (2)
C211	0.025 (3)	0.027 (3)	0.016 (3)	−0.004 (3)	0.008 (2)	0.000 (3)
C212	0.019 (3)	0.010 (2)	0.015 (3)	0.001 (2)	0.003 (2)	−0.001 (2)
C213	0.014 (3)	0.017 (3)	0.015 (3)	0.000 (2)	0.003 (2)	−0.001 (2)
C214	0.015 (3)	0.011 (2)	0.021 (3)	0.005 (2)	0.005 (2)	−0.001 (2)
C215	0.011 (3)	0.018 (3)	0.019 (3)	−0.002 (2)	0.002 (2)	0.000 (2)
C216	0.017 (3)	0.025 (3)	0.015 (3)	−0.003 (2)	−0.003 (2)	−0.003 (2)
C217	0.031 (4)	0.039 (4)	0.019 (3)	0.000 (3)	−0.001 (3)	−0.006 (3)
C218	0.038 (4)	0.038 (4)	0.026 (4)	0.015 (3)	−0.014 (3)	−0.008 (3)
C219	0.033 (4)	0.029 (4)	0.026 (4)	−0.012 (3)	−0.003 (3)	−0.007 (3)
C220	0.012 (3)	0.022 (3)	0.024 (3)	0.001 (2)	0.002 (2)	0.005 (3)
C221	0.017 (3)	0.017 (3)	0.018 (3)	0.002 (2)	0.005 (2)	0.001 (2)
C222	0.023 (3)	0.016 (3)	0.030 (4)	0.004 (2)	0.003 (3)	−0.001 (3)
C223	0.020 (3)	0.016 (3)	0.016 (3)	0.000 (2)	0.005 (2)	−0.003 (2)
C224	0.027 (3)	0.021 (3)	0.018 (3)	0.004 (2)	0.003 (3)	−0.006 (2)

### Geometric parameters (Å, º)

**Table:**

Ti1—O11	1.767 (4)	C119—H11J	0.98
Ti1—O13'	2.112 (9)	C119—H11K	0.98
Ti1—O13	2.157 (9)	C119—H11L	0.98
Ti1—Cl13	2.2451 (18)	C120—C121	1.389 (8)
Ti1—Cl11	2.2587 (18)	C120—H12B	0.95
Ti1—Cl12	2.2728 (17)	C121—C123	1.404 (7)
Ti2—O21	1.756 (4)	C121—C122	1.506 (8)
Ti2—O23	2.125 (4)	C122—H12C	0.98
Ti2—Cl21	2.2381 (19)	C122—H12D	0.98
Ti2—Cl23	2.2645 (19)	C122—H12E	0.98
Ti2—Cl22	2.2685 (18)	C123—C124	1.501 (7)
O11—C21	1.382 (6)	C124—H12F	0.98
O12—C214	1.383 (6)	C124—H12G	0.98
O12—H1	0.85 (3)	C124—H12H	0.98
O21—C11	1.382 (6)	C125—C126	1.497 (8)
O22—C114	1.379 (6)	C125—H12I	0.99
O22—H2	0.84 (3)	C125—H12J	0.99
O23—C125	1.471 (7)	C126—C127	1.520 (8)
O23—C128	1.473 (7)	C126—H12K	0.99
C11—C112	1.403 (7)	C126—H12L	0.99
C11—C12	1.413 (7)	C127—C128	1.489 (8)
C12—C17	1.394 (7)	C127—H12M	0.99
C12—C13	1.532 (8)	C127—H12N	0.99
C13—C15	1.535 (9)	C128—H12O	0.99
C13—C16	1.544 (9)	C128—H12P	0.99
C13—C14	1.553 (8)	C210—C212	1.418 (7)
C14—H14A	0.98	C210—C211	1.509 (7)
C14—H14B	0.98	C211—H21A	0.98
C14—H14C	0.98	C211—H21B	0.98
C15—H15A	0.98	C211—H21C	0.98
C15—H15B	0.98	C212—C213	1.492 (7)
C15—H15C	0.98	C213—C214	1.411 (7)
C16—H16A	0.98	C213—C223	1.415 (7)
C16—H16B	0.98	C214—C215	1.396 (7)
C16—H16C	0.98	C215—C220	1.408 (7)
C17—C18	1.400 (7)	C215—C216	1.557 (7)
C17—H17A	0.95	C216—C218	1.525 (8)
C18—C110	1.415 (7)	C216—C219	1.527 (8)
C18—C19	1.496 (7)	C216—C217	1.540 (8)
C19—H19A	0.98	C217—H21D	0.98
C19—H19B	0.98	C217—H21E	0.98
C19—H19C	0.98	C217—H21F	0.98
C21—C212	1.398 (7)	C218—H21G	0.98
C21—C22	1.411 (7)	C218—H21H	0.98
C22—C27	1.396 (7)	C218—H21I	0.98
C22—C23	1.538 (8)	C219—H21J	0.98
C23—C26	1.526 (8)	C219—H21K	0.98
C23—C25	1.536 (8)	C219—H21L	0.98
C23—C24	1.547 (8)	C220—C221	1.397 (7)
C24—H24A	0.98	C220—H22B	0.95
C24—H24B	0.98	C221—C223	1.402 (7)
C24—H24C	0.98	C221—C222	1.497 (7)
C25—H25A	0.98	C222—H22C	0.98
C25—H25B	0.98	C222—H22D	0.98
C25—H25C	0.98	C222—H22E	0.98
C26—H26A	0.98	C223—C224	1.521 (7)
C26—H26B	0.98	C224—H22F	0.98
C26—H26C	0.98	C224—H22G	0.98
C27—C28	1.398 (7)	C224—H22H	0.98
C27—H27A	0.95	O13—C228	1.478 (14)
C28—C210	1.402 (7)	O13—C225	1.481 (18)
C28—C29	1.512 (7)	C225—C226	1.46 (2)
C29—H29A	0.98	C225—H22A	0.99
C29—H29B	0.98	C225—H22I	0.99
C29—H29C	0.98	C226—C227	1.436 (19)
C110—C112	1.406 (7)	C226—H22J	0.99
C110—C111	1.511 (7)	C226—H22K	0.99
C111—H11A	0.98	C227—C228	1.541 (18)
C111—H11B	0.98	C227—H22L	0.99
C111—H11C	0.98	C227—H22M	0.99
C112—C113	1.506 (7)	C228—H22N	0.99
C113—C114	1.400 (7)	C228—H22O	0.99
C113—C123	1.409 (7)	O13'—C28'	1.465 (15)
C114—C115	1.415 (7)	O13'—C25'	1.506 (17)
C115—C120	1.396 (8)	C25'—C26'	1.474 (19)
C115—C116	1.549 (8)	C25'—H25D	0.99
C116—C119	1.533 (8)	C25'—H25E	0.99
C116—C118	1.534 (8)	C26'—C27'	1.451 (19)
C116—C117	1.538 (8)	C26'—H26D	0.99
C117—H11D	0.98	C26'—H26E	0.99
C117—H11E	0.98	C27'—C28'	1.481 (19)
C117—H11F	0.98	C27'—H27B	0.99
C118—H11G	0.98	C27'—H27C	0.99
C118—H11H	0.98	C28'—H28A	0.99
C118—H11I	0.98	C28'—H28B	0.99
			
O11—Ti1—O13'	87.9 (3)	C120—C121—C123	118.8 (5)
O11—Ti1—O13	87.4 (3)	C120—C121—C122	120.4 (5)
O11—Ti1—Cl13	131.13 (13)	C123—C121—C122	120.7 (5)
O13'—Ti1—Cl13	89.4 (3)	C121—C122—H12C	109.5
O13—Ti1—Cl13	81.6 (3)	C121—C122—H12D	109.5
O11—Ti1—Cl11	113.94 (13)	H12C—C122—H12D	109.5
O13'—Ti1—Cl11	80.9 (4)	C121—C122—H12E	109.5
O13—Ti1—Cl11	90.9 (4)	H12C—C122—H12E	109.5
Cl13—Ti1—Cl11	113.72 (7)	H12D—C122—H12E	109.5
O11—Ti1—Cl12	94.51 (12)	C121—C123—C113	118.8 (5)
O13'—Ti1—Cl12	173.9 (4)	C121—C123—C124	119.8 (5)
O13—Ti1—Cl12	174.5 (4)	C113—C123—C124	121.4 (5)
Cl13—Ti1—Cl12	93.26 (6)	C123—C124—H12F	109.5
Cl11—Ti1—Cl12	92.98 (7)	C123—C124—H12G	109.5
O21—Ti2—O23	87.15 (16)	H12F—C124—H12G	109.5
O21—Ti2—Cl21	122.51 (14)	C123—C124—H12H	109.5
O23—Ti2—Cl21	86.77 (12)	H12F—C124—H12H	109.5
O21—Ti2—Cl23	117.88 (14)	H12G—C124—H12H	109.5
O23—Ti2—Cl23	84.00 (12)	O23—C125—C126	104.6 (5)
Cl21—Ti2—Cl23	118.16 (7)	O23—C125—H12I	110.8
O21—Ti2—Cl22	95.09 (13)	C126—C125—H12I	110.8
O23—Ti2—Cl22	176.50 (13)	O23—C125—H12J	110.8
Cl21—Ti2—Cl22	94.27 (7)	C126—C125—H12J	110.8
Cl23—Ti2—Cl22	92.57 (7)	H12I—C125—H12J	108.9
C21—O11—Ti1	159.1 (3)	C125—C126—C127	102.3 (5)
C214—O12—H1	108 (4)	C125—C126—H12K	111.3
C11—O21—Ti2	163.3 (3)	C127—C126—H12K	111.3
C114—O22—H2	114 (4)	C125—C126—H12L	111.3
C125—O23—C128	108.1 (4)	C127—C126—H12L	111.3
C125—O23—Ti2	124.6 (3)	H12K—C126—H12L	109.2
C128—O23—Ti2	125.3 (3)	C128—C127—C126	103.3 (5)
O21—C11—C112	117.2 (5)	C128—C127—H12M	111.1
O21—C11—C12	119.9 (5)	C126—C127—H12M	111.1
C112—C11—C12	123.0 (5)	C128—C127—H12N	111.1
C17—C12—C11	114.7 (5)	C126—C127—H12N	111.1
C17—C12—C13	121.7 (5)	H12M—C127—H12N	109.1
C11—C12—C13	123.5 (5)	O23—C128—C127	106.3 (5)
C12—C13—C15	111.7 (5)	O23—C128—H12O	110.5
C12—C13—C16	109.5 (5)	C127—C128—H12O	110.5
C15—C13—C16	107.3 (6)	O23—C128—H12P	110.5
C12—C13—C14	112.5 (5)	C127—C128—H12P	110.5
C15—C13—C14	105.8 (5)	H12O—C128—H12P	108.7
C16—C13—C14	109.9 (5)	C28—C210—C212	119.1 (5)
C13—C14—H14A	109.5	C28—C210—C211	120.1 (5)
C13—C14—H14B	109.5	C212—C210—C211	120.8 (5)
H14A—C14—H14B	109.5	C210—C211—H21A	109.5
C13—C14—H14C	109.5	C210—C211—H21B	109.5
H14A—C14—H14C	109.5	H21A—C211—H21B	109.5
H14B—C14—H14C	109.5	C210—C211—H21C	109.5
C13—C15—H15A	109.5	H21A—C211—H21C	109.5
C13—C15—H15B	109.5	H21B—C211—H21C	109.5
H15A—C15—H15B	109.5	C21—C212—C210	119.3 (5)
C13—C15—H15C	109.5	C21—C212—C213	121.2 (5)
H15A—C15—H15C	109.5	C210—C212—C213	119.3 (5)
H15B—C15—H15C	109.5	C214—C213—C223	119.7 (5)
C13—C16—H16A	109.5	C214—C213—C212	120.4 (5)
C13—C16—H16B	109.5	C223—C213—C212	119.8 (5)
H16A—C16—H16B	109.5	O12—C214—C215	118.4 (5)
C13—C16—H16C	109.5	O12—C214—C213	119.5 (5)
H16A—C16—H16C	109.5	C215—C214—C213	122.1 (5)
H16B—C16—H16C	109.5	C214—C215—C220	116.0 (5)
C12—C17—C18	125.1 (5)	C214—C215—C216	122.4 (5)
C12—C17—H17A	117.5	C220—C215—C216	121.6 (5)
C18—C17—H17A	117.5	C218—C216—C219	107.7 (5)
C17—C18—C110	118.2 (5)	C218—C216—C217	106.2 (5)
C17—C18—C19	120.0 (5)	C219—C216—C217	109.7 (5)
C110—C18—C19	121.7 (5)	C218—C216—C215	111.1 (5)
C18—C19—H19A	109.5	C219—C216—C215	111.7 (5)
C18—C19—H19B	109.5	C217—C216—C215	110.2 (4)
H19A—C19—H19B	109.5	C216—C217—H21D	109.5
C18—C19—H19C	109.5	C216—C217—H21E	109.5
H19A—C19—H19C	109.5	H21D—C217—H21E	109.5
H19B—C19—H19C	109.5	C216—C217—H21F	109.5
O11—C21—C212	116.6 (4)	H21D—C217—H21F	109.5
O11—C21—C22	120.1 (5)	H21E—C217—H21F	109.5
C212—C21—C22	123.3 (5)	C216—C218—H21G	109.5
C27—C22—C21	114.9 (5)	C216—C218—H21H	109.5
C27—C22—C23	121.0 (5)	H21G—C218—H21H	109.5
C21—C22—C23	124.1 (5)	C216—C218—H21I	109.5
C26—C23—C25	108.0 (5)	H21G—C218—H21I	109.5
C26—C23—C22	110.9 (5)	H21H—C218—H21I	109.5
C25—C23—C22	109.3 (5)	C216—C219—H21J	109.5
C26—C23—C24	107.0 (5)	C216—C219—H21K	109.5
C25—C23—C24	109.9 (5)	H21J—C219—H21K	109.5
C22—C23—C24	111.7 (5)	C216—C219—H21L	109.5
C23—C24—H24A	109.5	H21J—C219—H21L	109.5
C23—C24—H24B	109.5	H21K—C219—H21L	109.5
H24A—C24—H24B	109.5	C221—C220—C215	124.0 (5)
C23—C24—H24C	109.5	C221—C220—H22B	118.0
H24A—C24—H24C	109.5	C215—C220—H22B	118.0
H24B—C24—H24C	109.5	C220—C221—C223	118.5 (5)
C23—C25—H25A	109.5	C220—C221—C222	120.7 (5)
C23—C25—H25B	109.5	C223—C221—C222	120.7 (5)
H25A—C25—H25B	109.5	C221—C222—H22C	109.5
C23—C25—H25C	109.5	C221—C222—H22D	109.5
H25A—C25—H25C	109.5	H22C—C222—H22D	109.5
H25B—C25—H25C	109.5	C221—C222—H22E	109.5
C23—C26—H26A	109.5	H22C—C222—H22E	109.5
C23—C26—H26B	109.5	H22D—C222—H22E	109.5
H26A—C26—H26B	109.5	C221—C223—C213	119.5 (5)
C23—C26—H26C	109.5	C221—C223—C224	120.2 (5)
H26A—C26—H26C	109.5	C213—C223—C224	120.3 (5)
H26B—C26—H26C	109.5	C223—C224—H22F	109.5
C22—C27—C28	124.5 (5)	C223—C224—H22G	109.5
C22—C27—H27A	117.8	H22F—C224—H22G	109.5
C28—C27—H27A	117.8	C223—C224—H22H	109.5
C27—C28—C210	119.0 (5)	H22F—C224—H22H	109.5
C27—C28—C29	119.9 (5)	H22G—C224—H22H	109.5
C210—C28—C29	121.2 (5)	C228—O13—C225	108.7 (9)
C28—C29—H29A	109.5	C228—O13—Ti1	120.5 (6)
C28—C29—H29B	109.5	C225—O13—Ti1	122.6 (9)
H29A—C29—H29B	109.5	C226—C225—O13	107.2 (11)
C28—C29—H29C	109.5	C226—C225—H22A	110.3
H29A—C29—H29C	109.5	O13—C225—H22A	110.3
H29B—C29—H29C	109.5	C226—C225—H22I	110.3
C112—C110—C18	119.1 (5)	O13—C225—H22I	110.3
C112—C110—C111	121.0 (5)	H22A—C225—H22I	108.5
C18—C110—C111	119.8 (5)	C227—C226—C225	106.7 (13)
C110—C111—H11A	109.5	C227—C226—H22J	110.4
C110—C111—H11B	109.5	C225—C226—H22J	110.4
H11A—C111—H11B	109.5	C227—C226—H22K	110.4
C110—C111—H11C	109.5	C225—C226—H22K	110.4
H11A—C111—H11C	109.5	H22J—C226—H22K	108.6
H11B—C111—H11C	109.5	C226—C227—C228	108.2 (11)
C11—C112—C110	119.9 (5)	C226—C227—H22L	110.1
C11—C112—C113	120.5 (5)	C228—C227—H22L	110.1
C110—C112—C113	119.5 (5)	C226—C227—H22M	110.1
C114—C113—C123	120.3 (5)	C228—C227—H22M	110.1
C114—C113—C112	119.8 (5)	H22L—C227—H22M	108.4
C123—C113—C112	119.7 (5)	O13—C228—C227	103.8 (9)
O22—C114—C113	120.4 (5)	O13—C228—H22N	111.0
O22—C114—C115	117.5 (5)	C227—C228—H22N	111.0
C113—C114—C115	122.1 (5)	O13—C228—H22O	111.0
C120—C115—C114	115.1 (5)	C227—C228—H22O	111.0
C120—C115—C116	122.2 (5)	H22N—C228—H22O	109.0
C114—C115—C116	122.7 (5)	C28'—O13'—C25'	107.6 (9)
C119—C116—C118	107.7 (5)	C28'—O13'—Ti1	125.0 (7)
C119—C116—C117	106.9 (5)	C25'—O13'—Ti1	126.7 (7)
C118—C116—C117	108.8 (5)	C26'—C25'—O13'	106.6 (10)
C119—C116—C115	111.4 (5)	C26'—C25'—H25D	110.4
C118—C116—C115	110.7 (5)	O13'—C25'—H25D	110.4
C117—C116—C115	111.1 (5)	C26'—C25'—H25E	110.4
C116—C117—H11D	109.5	O13'—C25'—H25E	110.4
C116—C117—H11E	109.5	H25D—C25'—H25E	108.6
H11D—C117—H11E	109.5	C27'—C26'—C25'	107.1 (12)
C116—C117—H11F	109.5	C27'—C26'—H26D	110.3
H11D—C117—H11F	109.5	C25'—C26'—H26D	110.3
H11E—C117—H11F	109.5	C27'—C26'—H26E	110.3
C116—C118—H11G	109.5	C25'—C26'—H26E	110.3
C116—C118—H11H	109.5	H26D—C26'—H26E	108.6
H11G—C118—H11H	109.5	C26'—C27'—C28'	107.9 (11)
C116—C118—H11I	109.5	C26'—C27'—H27B	110.1
H11G—C118—H11I	109.5	C28'—C27'—H27B	110.1
H11H—C118—H11I	109.5	C26'—C27'—H27C	110.1
C116—C119—H11J	109.5	C28'—C27'—H27C	110.1
C116—C119—H11K	109.5	H27B—C27'—H27C	108.4
H11J—C119—H11K	109.5	O13'—C28'—C27'	106.1 (10)
C116—C119—H11L	109.5	O13'—C28'—H28A	110.5
H11J—C119—H11L	109.5	C27'—C28'—H28A	110.5
H11K—C119—H11L	109.5	O13'—C28'—H28B	110.5
C121—C120—C115	124.8 (5)	C27'—C28'—H28B	110.5
C121—C120—H12B	117.6	H28A—C28'—H28B	108.7
C115—C120—H12B	117.6		
			
O13'—Ti1—O11—C21	141.2 (10)	C115—C120—C121—C123	−1.6 (8)
O13—Ti1—O11—C21	130.3 (10)	C115—C120—C121—C122	178.6 (5)
Cl13—Ti1—O11—C21	53.8 (10)	C120—C121—C123—C113	1.5 (8)
Cl11—Ti1—O11—C21	−139.8 (9)	C122—C121—C123—C113	−178.7 (5)
Cl12—Ti1—O11—C21	−44.5 (9)	C120—C121—C123—C124	−180.0 (5)
O23—Ti2—O21—C11	166.2 (13)	C122—C121—C123—C124	−0.2 (8)
Cl21—Ti2—O21—C11	81.9 (13)	C114—C113—C123—C121	0.2 (8)
Cl23—Ti2—O21—C11	−112.1 (13)	C112—C113—C123—C121	176.2 (5)
Cl22—Ti2—O21—C11	−16.5 (13)	C114—C113—C123—C124	−178.2 (5)
Ti2—O21—C11—C112	−63.3 (14)	C112—C113—C123—C124	−2.3 (7)
Ti2—O21—C11—C12	116.5 (12)	C128—O23—C125—C126	−20.2 (6)
O21—C11—C12—C17	−178.2 (5)	Ti2—O23—C125—C126	174.8 (3)
C112—C11—C12—C17	1.6 (8)	O23—C125—C126—C127	36.0 (6)
O21—C11—C12—C13	−1.6 (8)	C125—C126—C127—C128	−38.4 (7)
C112—C11—C12—C13	178.2 (5)	C125—O23—C128—C127	−4.2 (7)
C17—C12—C13—C15	−8.9 (9)	Ti2—O23—C128—C127	160.6 (4)
C11—C12—C13—C15	174.8 (6)	C126—C127—C128—O23	26.4 (7)
C17—C12—C13—C16	109.8 (6)	C27—C28—C210—C212	−1.8 (8)
C11—C12—C13—C16	−66.5 (7)	C29—C28—C210—C212	178.9 (5)
C17—C12—C13—C14	−127.7 (6)	C27—C28—C210—C211	176.3 (5)
C11—C12—C13—C14	55.9 (8)	C29—C28—C210—C211	−2.9 (8)
C11—C12—C17—C18	−0.3 (8)	O11—C21—C212—C210	178.6 (4)
C13—C12—C17—C18	−177.0 (5)	C22—C21—C212—C210	0.4 (8)
C12—C17—C18—C110	−0.9 (9)	O11—C21—C212—C213	3.2 (7)
C12—C17—C18—C19	177.7 (5)	C22—C21—C212—C213	−175.1 (5)
Ti1—O11—C21—C212	−43.0 (12)	C28—C210—C212—C21	1.4 (7)
Ti1—O11—C21—C22	135.3 (8)	C211—C210—C212—C21	−176.7 (5)
O11—C21—C22—C27	−179.8 (4)	C28—C210—C212—C213	176.9 (5)
C212—C21—C22—C27	−1.6 (7)	C211—C210—C212—C213	−1.2 (7)
O11—C21—C22—C23	−3.1 (8)	C21—C212—C213—C214	−87.0 (6)
C212—C21—C22—C23	175.1 (5)	C210—C212—C213—C214	97.5 (6)
C27—C22—C23—C26	−5.7 (7)	C21—C212—C213—C223	97.2 (6)
C21—C22—C23—C26	177.8 (5)	C210—C212—C213—C223	−78.3 (6)
C27—C22—C23—C25	113.2 (6)	C223—C213—C214—O12	177.5 (5)
C21—C22—C23—C25	−63.3 (7)	C212—C213—C214—O12	1.6 (7)
C27—C22—C23—C24	−124.9 (5)	C223—C213—C214—C215	−1.6 (8)
C21—C22—C23—C24	58.6 (7)	C212—C213—C214—C215	−177.5 (5)
C21—C22—C27—C28	1.1 (7)	O12—C214—C215—C220	−176.3 (5)
C23—C22—C27—C28	−175.6 (5)	C213—C214—C215—C220	2.8 (8)
C22—C27—C28—C210	0.5 (8)	O12—C214—C215—C216	3.6 (8)
C22—C27—C28—C29	179.8 (5)	C213—C214—C215—C216	−177.3 (5)
C17—C18—C110—C112	0.8 (8)	C214—C215—C216—C218	176.4 (5)
C19—C18—C110—C112	−177.7 (5)	C220—C215—C216—C218	−3.8 (7)
C17—C18—C110—C111	176.3 (5)	C214—C215—C216—C219	−63.4 (7)
C19—C18—C110—C111	−2.2 (8)	C220—C215—C216—C219	116.5 (6)
O21—C11—C112—C110	178.1 (4)	C214—C215—C216—C217	58.9 (7)
C12—C11—C112—C110	−1.6 (8)	C220—C215—C216—C217	−121.3 (5)
O21—C11—C112—C113	1.9 (7)	C214—C215—C220—C221	−1.1 (8)
C12—C11—C112—C113	−177.8 (5)	C216—C215—C220—C221	179.0 (5)
C18—C110—C112—C11	0.4 (8)	C215—C220—C221—C223	−1.8 (8)
C111—C110—C112—C11	−175.0 (5)	C215—C220—C221—C222	179.9 (5)
C18—C110—C112—C113	176.6 (5)	C220—C221—C223—C213	3.0 (8)
C111—C110—C112—C113	1.2 (7)	C222—C221—C223—C213	−178.6 (5)
C11—C112—C113—C114	−85.7 (6)	C220—C221—C223—C224	−179.2 (5)
C110—C112—C113—C114	98.1 (6)	C222—C221—C223—C224	−0.9 (8)
C11—C112—C113—C123	98.3 (6)	C214—C213—C223—C221	−1.4 (8)
C110—C112—C113—C123	−77.9 (6)	C212—C213—C223—C221	174.4 (5)
C123—C113—C114—O22	177.3 (5)	C214—C213—C223—C224	−179.2 (5)
C112—C113—C114—O22	1.3 (7)	C212—C213—C223—C224	−3.3 (7)
C123—C113—C114—C115	−2.1 (8)	C228—O13—C225—C226	14.4 (16)
C112—C113—C114—C115	−178.0 (5)	Ti1—O13—C225—C226	162.9 (10)
O22—C114—C115—C120	−177.4 (5)	O13—C225—C226—C227	−23.0 (19)
C113—C114—C115—C120	2.0 (8)	C225—C226—C227—C228	22.7 (19)
O22—C114—C115—C116	2.9 (8)	C225—O13—C228—C227	−0.7 (13)
C113—C114—C115—C116	−177.7 (5)	Ti1—O13—C228—C227	−149.9 (14)
C120—C115—C116—C119	−3.9 (8)	C226—C227—C228—O13	−13.6 (16)
C114—C115—C116—C119	175.9 (5)	C28'—O13'—C25'—C26'	−0.7 (14)
C120—C115—C116—C118	115.9 (6)	Ti1—O13'—C25'—C26'	−171.3 (10)
C114—C115—C116—C118	−64.3 (7)	O13'—C25'—C26'—C27'	13.6 (18)
C120—C115—C116—C117	−123.0 (6)	C25'—C26'—C27'—C28'	−21 (2)
C114—C115—C116—C117	56.7 (7)	C25'—O13'—C28'—C27'	−12.1 (14)
C114—C115—C120—C121	−0.2 (8)	Ti1—O13'—C28'—C27'	158.7 (16)
C116—C115—C120—C121	179.6 (5)	C26'—C27'—C28'—O13'	20.9 (18)

### Hydrogen-bond geometry (Å, º)

**Table:**

*D*—H···*A*	*D*—H	H···*A*	*D*···*A*	*D*—H···*A*
O12—H1···Cl12^i^	0.85 (3)	2.62 (4)	3.333 (4)	143 (5)
O22—H2···Cl22^i^	0.84 (3)	2.59 (4)	3.350 (4)	153 (5)
C125—H12*J*···O22	0.99	2.63	3.324 (7)	127
C228—H22*O*···Cl11	0.99	2.98	3.538 (14)	117
C25′—H25*D*···O12	0.99	2.25	3.231 (16)	170
C28′—H28*A*···Cl13	0.99	2.88	3.448 (16)	117

Symmetry code: (i) *x*, *y*+1, *z*.
